# A giant Brunner’s gland hamartoma being treated as a pedunculated polyp: a case report

**DOI:** 10.1186/s12876-019-1074-1

**Published:** 2019-08-23

**Authors:** Lizhi Yi, Zhengyu Cheng, Huarong Qiu, Jianjun Yang, Tao Wang, Ke Liu

**Affiliations:** 1 Department of Gastroenterology, People’s Hospital of Leshan, 238 Baita Street, Leshan, Sichuan 614000 People’s Republic of China; 2 Department of pathology, People’s Hospital of Leshan, Leshan, City, 614000 Sichuan province People’s Republic of China; 3 Department of radiology, People’s Hospital of Leshan, Leshan, City, 614000 Sichuan province People’s Republic of China

**Keywords:** Brunner’s gland hamartoma, Severe anemia, Hot snare polypectomy, Endoscopy, Case report

## Abstract

**Background:**

With the development and application of endoscopic technology, most pedunculated polyps can be absolutely resected with a complete specimen by hot snare polypectomy (HSP). Brunner’s gland hamartoma (BGH) is a rare benign small bowel tumor. The majority of BGH measuring about 2 cm in diameter, rarely larger than 5 cm. Most patients are asymptomatic, some may present with gastrointestinal hemorrhage or intestinal obstruction. Symptomatic larger lesions leading to bleeding or obstruction should be excised either endoscopically or surgically. Whether it is safe and effective that removing a BGH measuring about 7 cm by HSP is not known.

**Case presentation:**

Here, we reported a rare case of a proximal duodenum pedunculated mass measuring about 7 cm which was responsible for the patient’s severe anemia. we treated it as a pedunculated polyp. After being pretreated the stalk with an endoloop which was placed around the base of the mass to prevent post-polypectomy bleeding (PPB), the pedunculated BGH was removed by HSP completely. The stalk of the mass was negative. We achieved a curative resection.

**Conclusion:**

It is a safe and effective for our patient to treat the pedunculated BGH measuring about 7 cm as a pedunculated polyp and remove it by HSP. And future prospective studies in larger cohorts are needed to confirm it.

## Background

For pedunculated polyps, most can be easily removed completely by hot snare polypectomy (HSP). It can not only allow us to obtain a complete specimen, but also can achieve a curative resection [1]. Is this strategy applicable to other pedunculated masses, of which even the size is about 7 cm? Brunner’s gland hamartoma (BGH) is a benign tumor of the duodenum arising from the Brunner’s glands [2]. Most patients are asymptomatic, but some may present with common gastrointestinal symptoms such as bleeding, nausea, vomiting, and chronic abdominal pain [3]. Brunner’s gland hamartoma can be treated either by endoscopic or surgical excision [4]. To our knowledge, up to now, only 4 cases of endoscopic resection of BGH larger than 7 cm have been reported [5–8]. In two cases, the BGH was removed by endoscopic submucosal dissection (ESD) and endoscopic mucosal resection (EMR) respectively [5, 6]. In the other two cases being reported 13 years and 18 years ago respectively, neither of them pretreated the stalk, which was very important to the strategy for the treatment of pedunculated mass, to prevent the main adverse event post-polypectomy bleeding (PPB) [7, 8]. Whether it is safe and effective that removing a 7 cm BGH by HSP is not known.

Here, we report a rare case that a pedunculated BGH measuring about 7 cm in proximal duodenum, causing severe anemia, was treated as a pedunculated polyp and being successfully removed by hot snare polypectomy (HSP) in a curable way.

## Case presentation

**Fig. 1:**
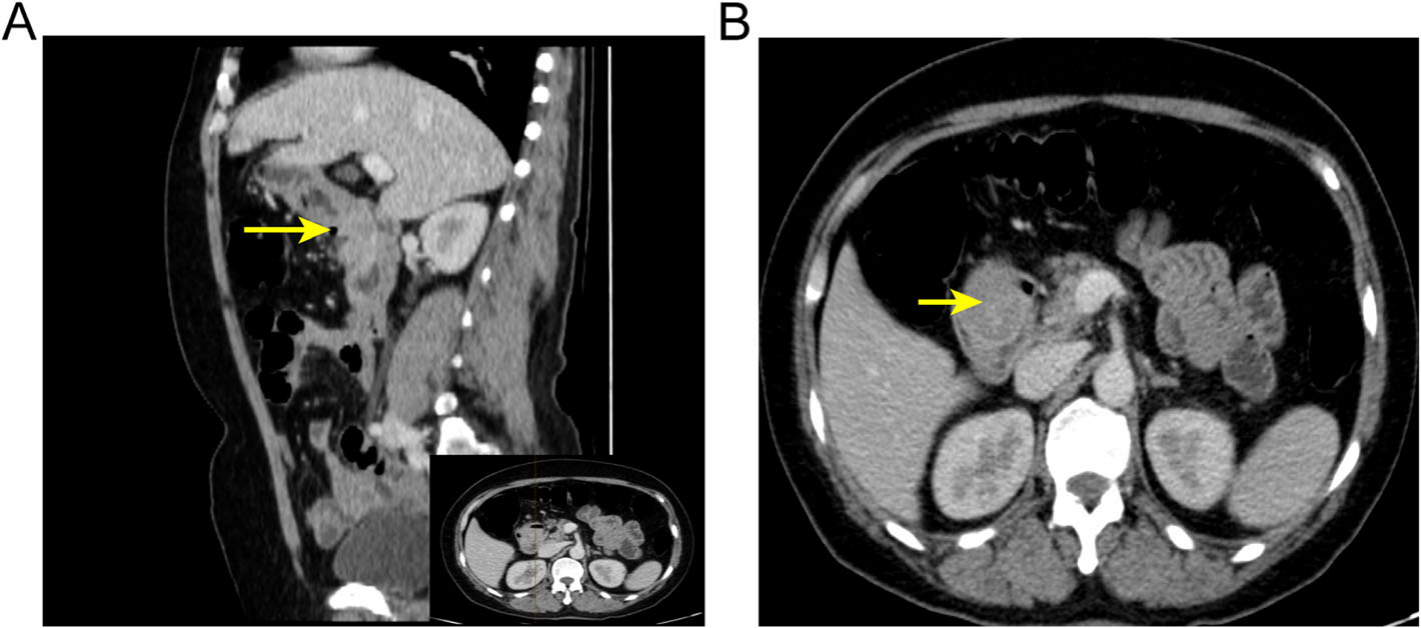
(**a**) Contrast-enhanced sagittal and (**b**) axial computed tomography scan shows a large polypoid mass in the duodenal bulb (arrows)

**Fig. 2:**
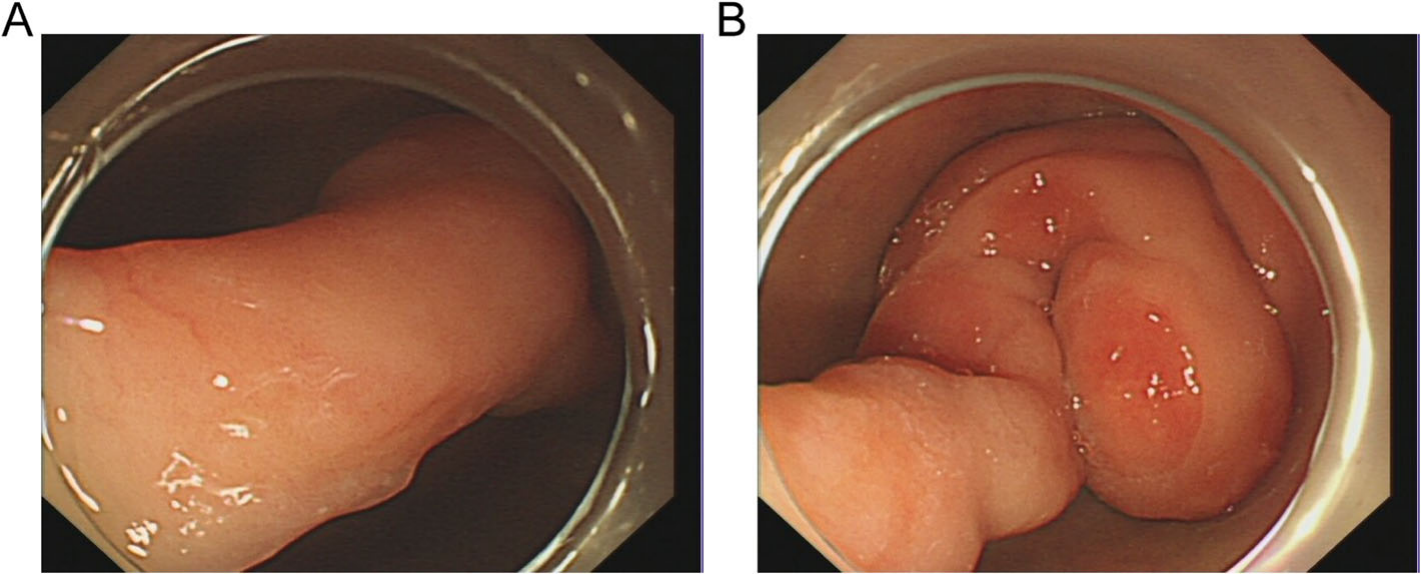
Esophagogastroduodenoscopy shows a tortuous pedunculated mass occupying the lumen of the duodenal bulb. (**a**) The long and thick pedicle of the tumor. (**b**) The tumor is coiled around the duodenal bulb

**Fig. 3:**
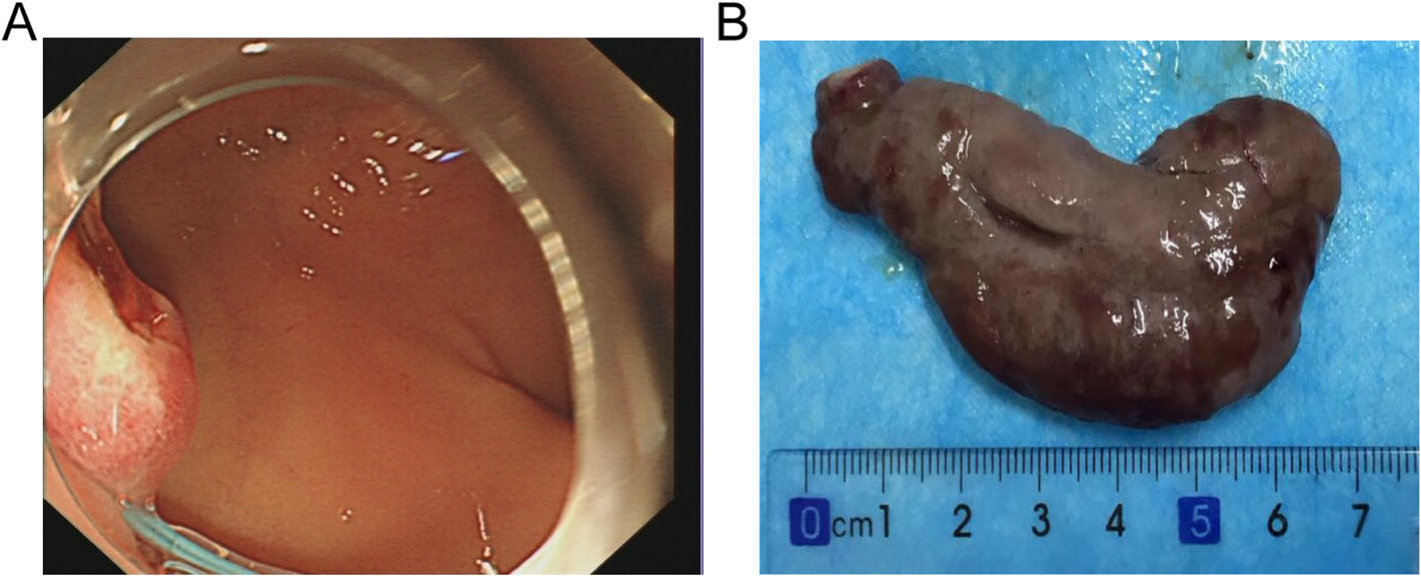
(**a**) The appearance after polypectomy (**b**) The complete endoscopic resection specimen showed a large duodenal lesion measuring 7 × 3 × 1.6 cm^3^

**Fig. 4:**
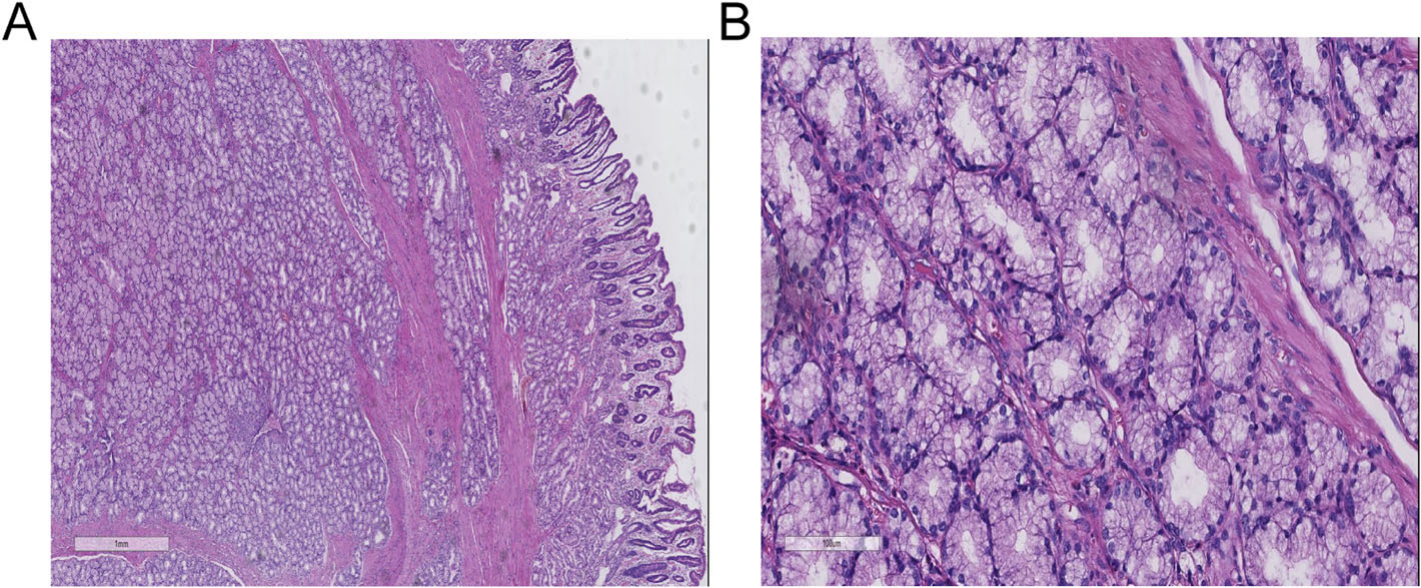
Microscopic pictures. (**a**) Light microscopy showed hyperplastic lobules of proliferating Brunner’s glands separated by fibrous. H&E staining, scale bar: 1 mm. (**b**) Brunner’s gland hyperplasia composed of variable size of Brunner’s glands can be observed. H&E staining, scale bar: 100 μm

A 63-year-old woman complained with a 20-days history of melena, epigastric pain and fatigue. A single episode of melena had occurred 5 months ago but no further investigation was performed. The patient has no medical history of taking nonsteroidal anti-inflammatory drugs, including no family history of cancer or surgery. Before being referred to our hospital, she initially presented to a clinic and was informed a grant lesion by esophagogastroduodenoscopy. At physical examination of the woman, pallor, sensitivity in the epigastric zone were noted. Vital signs (temperature 36.6 C, pulse 91 beats/minute, pressure of 125/75 mmHg, oximetry saturation100%) were normal. Melena was detected in the rectal examination. Hematological test showed a severe anemia with 32 g/L hemoglobin (normal range, 115-150 g/L), hematocrit 11.7% (mean corpuscular volume 66 fL, mean corpuscular hemoglobin 18.1 pg). Blood iron level was 6.8 ng/ml (normal range, 10-291 ng/mL). Intravenous iron supplement was offered to this lady. *Helicobacter pylori* test was negative. Due to her microcytic anemia, a colonoscopy was performed and came out to be negative. After 2-unit blood transfusions of erythrocyte concentrates, the woman felt better and refused more transfusions. Computed tomography analysis showed a giant mass near the proximal duodenum (Fig. 1a and b). Esophagogastroduodenoscopy showed a giant pedunculated mass with a congested and erosive appearance arising from the duodenal bulb (Fig. 2a and b). No difference was observed between most appearance of the mass and the duodenal normal mucosa. The tumor was not the same as the pedunculated polyp we treated as usual. Because the mass was suspected of being responsible for the patient’s severe anemia, removing the mass was necessary. For the long stalk, the mass could be completely removed by HSP easily like a pedunculated polyp. And a pedunculated specimen could be easily obtained for a further histological examination. We thought the strategy for pedunculated polyps was applicable to this pedunculated mass. After being informed of all approaches to treating this mass, including the risk of endoscopic perforation or bleeding, the patient consented to remove this mass by HSP. We treated this mass as a pedunculated polyp, pretreating the stalk with an endoloop which was placed around the base of the mass to prevent post-polypectomy bleeding (PPB). Then the pedunculated mass was removed by HSP easily. In order to extracting the removed specimen, we used the snare to pull part of the mass into the transparent cap by trapping the smaller end of the mass. Then the specimen was pulled out following the endoscope and a complete specimen was obtained (Fig. 3a). The process was simple and effective, and usually took less time than ESD or EMR. The stalk of the specimen was dealt with carefully for histological examination. The gross endoscopic resection specimen showed a large duodenal lesion measuring 7 × 3 × 1.6 cm^3^ (Fig. 3b). The resected pedunculated mass was histologically confirmed a giant Brunner’s gland hamartoma (Fig. 4a and b). And the stalk was negative. Epigastric pain and melena of the patient were resolved after the endoscopic treatment and two weeks later, her hemoglobin rose up to 82 g/L. Three months later, her hemoglobin was normalized and no evidence of recurrence or tumor remnants were found by a repeat esophagogastroduodenoscopy.

## Discussion and conclusion

Brunner’s glands were first described by Brunner in 1688 [9]. They locate in duodenal submucosa and secrete some alkaline fluid which can avoid gastric acid eroding duodenal epithelium [2]. The benign hyperplastic lesions of Brunner glands are described as Brunner gland hyperplastic nodules/polyps or Brunner gland hamartomas [10]. As a rule, lesions smaller than 2 cm defined as Brunner’s gland hyperplastic nodules/polyps, while lesions larger than 2 cm termed Brunner’s gland hamartoma [11]. The pathogenesis of BGH remains obscure. Chronic pancreatitis and *Helicobacter pylori* infection are suspected to be involved [2, 6]. Because BGH was usually covered with normal mucosa, the value of pinch biopsies might be limited. Endoscopic ultrasound (EUS) may be helpful in the diagnosis and treatment of BGH [12]. In our case, taking into account that a complete specimen for histological examination could be obtained easily by HSP, we did not perform a EUS. However, we believe that the endoscopic resection should been much safer if we had performed a EUS which could offer us some information about the nature of the mass and submucosal vascular. Usually, asymptomatic small Brunner’s gland hamartomas require no treatment. While symptomatic and larger lesions leading to bleeding or obstruction should be excised either endoscopically or surgically [11].

Endoscopic treatments for large Brunner’s hamartomas with relatively low complication and mortality rates is effective and safe [5, 6]. But not all the large BGH could be removed endoscopically. As Table 1 shows, in reported cases of Brunner’s gland hamartomas larger than 5 cm, all the exophytic BGH and sessile BGH are removed surgically, while Endoscopic resection was only performed in the pedunculated BGH. In addition, surgical treatment was also chosen in the pedunculated mass in the following cases: [1] Malignancy of the mass can not be excluded; [2] some emergency complications like intussusception; [3] The mass was too large, or the stalk was too thick to be removed endoscopically. Therefore, preoperative diagnosis and endoscopic evaluation are important to proper selection of the interventional approach.

**Table 1:** The characteristics of Brunner’s gland hamartomas larger than 5 cm and interventional approaches in reported cases

Reference	Size (cm)	Growth pattern	PeduncuLated or sessile	Reasons for choosing different interventional approaches	The interventional approach
[13]	8 × 10	exophytic type	not applicable	Not given clearly	Surgery
[14]	5 X 6	exophytic type	not applicable	Malignancy could not be excluded.	Surgery
[15]	7.9	exophytic type	not applicable	Not given clearly	Surgery
[16]	5–6	exophytic type	not applicable	Not given clearly	Surgery
[17]	6	exophytic type	not applicable	Not given clearly	Surgery
[18]	6.6 × 4.5	exophytic type	not applicable	Not given clearly	Surgery
[19]	5.5 × 3.3× 2.2	intraluminal type	sessile	Malignancy could not be excluded	Surgery
[20]	5.5	intraluminal type	sessile	unknown nature of the mass	Surgery
[21]	10.5	intraluminal type	sessile	Not given clearly	Surgery
[22]	7.3 × 3.4× 2.9	intraluminal type	sessile	The suspicion for malignancy was high	Surgery
[23]	7.5 × 6.5× 6.5	intraluminal type	sessile	Not given clearly	Surgery
[24]	10 × 6 × 8	intraluminal type	sessile	Not given clearly	Surgery
[25]	12 × 10 × 8	intraluminal type	sessile	Not given clearly	Surgery
[3]	8 × 4 × 8	intraluminal type	sessile	uncertain malignant potential	Surgery
[26]	6 × 2.4	intraluminal type	pedunculated	Not given clearly	Surgery
[27]	6 × 3	intraluminal type	pedunculated	Not given clearly	Surgery
[28]	5 × 3、6 × 3.5	intraluminal type	pedunculated	Not given clearly	Surgery
[29]	6 × 4	intraluminal type	pedunculated	the large size of the tumor	Surgery
[30]	7.3 × 3.4 × 2.9	intraluminal type	pedunculated	Not given clearly	Surgery
[31]	8	intraluminal type	pedunculated	Not given clearly	Surgery
[32]	3 × 10	intraluminal type	pedunculated	intussusception	Surgery
[33]	10–12	intraluminal type	pedunculated	suspected malignant transformation	Surgery
[2]	10 × 2 × 1.5	intraluminal type	peduncuLated	large size and the difficulty in gaining access to the head of the polyp for snaring.	Surgery
[34]	6.4 × 3	intraluminal type	pedunculated	The stalk was too thick	Surgery
[35]	5.5 × 4.2 × 4.3	intraluminal type	pedunculated	The polyp was too large	Surgery
[36]	6x5x3	intraluminal type	pedunculated	Not given clearly	Surgery
[5]	7 × 2	intraluminal type	pedunculated	Not given clearly	Endoscopic polypectomy
[6]	9.3 × 2	intraluminal type	pedunculated	Not given clearly	Endoscopic polypectomy
[7]	7	intraluminal type	pedunculated	Not given clearly	Endoscopic polypectomy
[8]	10.5	intraluminal type	pedunculated	Not given clearly	Endoscopic polypectomy
[37]	6.5 × 4 × 4	intraluminal type	pedunculated	no invasion andintraluminal type	Endoscopic polypectomy
[38]	6 × 0.9	intraluminal type	pedunculated	For both the diagnosis and the treatment.	Endoscopic polypectomy
[39]	6.0 × 0.4 × 0.2	intraluminal type	pedunculated	Not given clearly	Endoscopic polypectomy
[40]	5	intraluminal type	pedunculated	Not given clearly	Endoscopic polypectomy

Large pedunculated polyps have an increased risk of PPB because of the presence of a large blood vessel within the stalk [41]. Pretreatment of the stalk is a recommended method to prevent it [1]. In our case, the giant pedunculated mass measuring about 7 cm was suspected of being responsible for the patient’s severe anemia. We treated it as a pedunculated polyp. After being pretreated the stalk to prevent PPB, the pedunculated BGH was removed by HSP completely. The stalk of the mass was negative. We achieve a curative resection. For our patient, removing a pedunculated by HSP is safe and effective. Future prospective studies in larger cohorts are necessary for further verification.

## Data Availability

Not applicable.

## Abbreviations

BGH: Brunner’s gland hamartoma
EMR: Endoscopic mucosal resection
ESD: Endoscopic submucosal dissection
EUS: Endoscopic ultrasound
HSP: Hot snare polypectomy
PPB: Post-polypectomy bleeding
